# Resting-state cortical activity, biomarkers and functional performance identify distinct biopsychosocial phenotypes in young adults with chronic postsurgical pain

**DOI:** 10.3389/fpain.2026.1814480

**Published:** 2026-04-29

**Authors:** Guillermo Ceniza-Bordallo, Ziyan Wu, Caitlin Curry, Christine B. Sieberg

**Affiliations:** 1 Center for Health Outcomes and Interdisciplinary Research, Department of Psychiatry, Massachusetts General Hospital, Boston, MA, United States; 2 Department of Psychiatry, Harvard Medical School, Boston, MA, United States; 3 Division of Adolescent and Young Adult Medicine, Department of Pediatrics, Boston Children’s Hospital, Harvard Medical School, Boston, MA, United States

**Keywords:** biomarkers, brain alterations, chronic postsurgical pain, physical functioning, young adults

## Abstract

**Background:**

Chronic postsurgical pain (CPSP) is a prevalent and disabling condition in young adults (AYAs) yet marked variability in symptoms and functional impact is poorly explained by pain severity alone. This study aimed to (1) compare psychological symptoms, physical performance, hair cortisol concentration, and resting-state cortical hemodynamic activity between AYAs with CPSP and healthy controls; (2) examine associations among these variables within the CPSP group; and (3) derive clinically meaningful CPSP phenotypes anchored in physical performance using hierarchical clustering.

**Methods:**

In this cross-sectional study, AYAs aged 18–30 years with CPSP and healthy controls were enrolled. Psychological symptoms and pain interference were assessed using standardized self-report measures. Physical performance was evaluated using the 1 min sit-to-stand test, with concurrent physiological responses to exertion. Resting-state cortical hemodynamic activity was assessed using functional near-infrared spectroscopy (fNIRS).

**Results:**

A total of 67 AYAs (33 with CPSP and 34 healthy controls) were included. Compared with healthy controls, AYAs with CPSP reported significantly greater pain interference and exhibited dysregulated physiological responses to exertion, despite comparable objective physical performance. Within the CPSP group, psychological distress, hair cortisol concentration, physical performance, and resting-state prefrontal and somatosensory cortex (SMC) (S1) activity were significantly interrelated. Clustering analyses identified two distinct CPSP phenotypes, low functioning and high functioning, which differed in pain catastrophizing, autonomic reactivity, and resting state SMC (S1) activity.

**Conclusions:**

CPSP in AYAs is associated with distinct autonomic, neuroendocrine, and cortical signatures that co-vary with functional performance. Integrating physiological biomarkers and resting-state neuroimaging with performance-based assessments may improve mechanistic understanding and phenotyping of CPSP.

## 1 Introduction

Chronic pain represents a major public health concern in adolescents and young adults (AYAs), with prevalence estimates ranging from 20% to 35% (1). Chronic pain in this population is associated with substantial impairments in daily functioning, including difficulties participating in school, reduced engagement in physical activity, sleep problems, and heightened emotional distress (2–5).

Chronic postsurgical pain (CPSP) is a secondary chronic pain condition defined as persistent pain lasting at least three months after surgery in the absence of other causes, affecting approximately 10%–20% of AYAs following major surgical procedures (6, 7). CPSP is associated with higher rates reported after orthopedic, thoracic, and abdominal surgeries (8–10). In AYAs, CPSP is associated with increased healthcare utilization, reduced engagement in physical activity, heightened emotional distress, and difficulties resuming typical school, work, and recreational activities (7, 8, 11, 12).

Importantly, the transition from adolescence to young adulthood represents a sensitive developmental window during which the emergence of chronic pain may have particularly detrimental and long-lasting consequences (13, 14). CPSP during this period may initiate or exacerbate neurobiological and psychosocial processes that increase vulnerability to persistent pain, functional disability, and mood and anxiety disorders lasting into adulthood (4, 15). Early pain-related disruptions have also been linked to sustained reductions in physical activity and alterations in brain circuits involved in pain modulation and emotional regulation, potentially reinforcing maladaptive trajectories across the lifespan (14, 16, 17).

Chronic stress is a pivotal factor in the development and persistence of pain during adolescence and the transition to young adulthood (18). Prolonged stress exposure can dysregulate the hypothalamic–pituitary–adrenal (HPA) axis, lead to aberrant cortisol secretion patterns, and increase vulnerability to mental illnesses and chronic pain (19). Such dysregulation is associated with disruptions in prefrontal and limbic circuitry, impairing emotional regulation and nociceptive control (20). Importantly, higher physical functioning and regular activity are linked to healthier stress physiology—more stable cortisol rhythms, improved autonomic balance, and better prefrontal regulation—suggesting that physical functioning may buffer the impact of chronic stress on the brain and emotional health (21, 22).

A growing body of evidence indicates that higher physical functioning—through greater daily activity and preserved functional capacity—has a broad regulatory influence on the emotional and neurocognitive systems involved in chronic pain (23–30). Regular physical activity is associated with lower levels of catastrophizing, depressive symptoms, anxiety, and pain interference, partly through its effects on stress physiology, endogenous analgesia, and emotional regulation pathways (26, 30–35).

Exercise stimulates the release of neurotrophic factors such as BDNF, modulates inflammatory signaling, enhances autonomic balance, and improves affective regulation through increased prefrontal engagement and reduced limbic hyperreactivity (36). Neuroimaging studies consistently show that children (37) and adults (38) who show higher physical fitness or greater habitual activity display more efficient functioning of prefrontal regions—particularly the lateral and medial PFC—which support cognitive control, emotion regulation, and top-down modulation of nociceptive input. Moreover, higher physical functioning has been linked in adults (38) to stronger prefrontal–periaqueductal gray (PAG) and prefrontal–somatosensory connectivity, more coherent resting-state organization within the PFC, and reduced spontaneous dysregulation of hemodynamic activity.

These neurocognitive adaptations may enable individuals to regulate negative emotions more effectively, buffer the impact of pain-related stressors, and maintain stable behavioral patterns that prevent further disability. Thus, children and adolescents with chronic pain who sustain higher physical functioning may not only present with better emotional functioning, but also with prefrontal cortical patterns indicative of more adaptive neural regulation (39).

Together, these findings suggest that physical functioning may contribute to pain resilience through interconnected psychological and cerebral mechanisms; however, this hypothesis has not yet been examined in AYAs with CPSP using integrated psychological, physiological, and neurobiological measures. Current studies largely assess these domains in isolation, limiting our ability to identify mechanistically informed phenotypes that may guide targeted rehabilitation strategies.

Based on these considerations, the aims of this study were to: [1] compare psychological symptoms, physical performance, hair cortisol concentration, and resting-state cortical hemodynamic activity, between AYAs with CPSP and healthy controls; [2] examine associations between pain interference, psychological symptoms, physical performance, hair cortisol concentration, and resting state cortical hemodynamic activity in AYAs with CPSP and; as exploratory aim [3] to explore preliminary biopsychosocial phenotypes of physical performance using a clustering approach that integrates pain interference, psychological symptoms, hair cortisol concentration, and resting state cortical hemodynamic activity in AYAs with CPSP.

We hypothesize that AYAs with CPSP will show higher psychological symptoms, lower physical performance, higher hair cortisol concentration, and greater magnitude of resting-state cortical hemodynamic activity as compared to healthy controls. Additionally, we hypothesize that in AYAs with CPSP, higher pain interference, higher psychological symptoms, elevated hair cortisol concentration, and reduced physical performance will be associated with greater resting-state cortical hemodynamic activity. Finally, we hypothesize that distinct physical performance clusters will emerge, characterized by statistically and clinically meaningful differences in pain interference, psychological symptoms, hair cortisol concentration, physical performance and fitness, and resting state cortical hemodynamic activity.

## 2 Methods

### 2.1 Design

This observational cross-sectional study was conducted in accordance with the Declaration of Helsinki and received approval from the Institutional Review Boards of the participating institutions. The design and reporting of this study followed the Strengthening the Reporting of Observational Studies in Epidemiology (STROBE) guidelines for observational research (40).

### 2.2 Eligibility criteria

Given that the age range used to define AYAs varies across the literature (1, 41), AYAs aged 18 to 30 years were included in this study, adopting this operational definition. AYAs were able to provide informed consent and had sufficient proficiency in English to complete study procedures. Individuals were excluded if they reported a history of severe neurological or psychiatric disorders, recent traumatic brain injury, current or recent illicit substance use, or use of medications known to substantially affect cortical activity or cardiovascular responses (e.g., benzodiazepines, antipsychotics, beta-blockers, or stimulant medications).

Participants were subsequently classified into one of two groups based on their chronic pain status. AYAs in the CPSP group were required to report persistent pain related to a surgical procedure that had lasted for at least three months following surgery, consistent with established criteria for CPSP (42). Healthy control AYAs were required to report no current or prior history of chronic pain and no history of surgical procedures or medical conditions that would contraindicate participation in physical or neurophysiological testing. Tooth extraction, without general anesthesia, was permitted if it had occurred more than two years prior to enrollment.

### 2.3 Variables

#### 2.3.1 Pain intensity

Pain intensity was assessed using a numerical rating scale (NRS). Participants were asked to rate their average pain intensity over the past seven days on an 11-point scale ranging from 0 (“no pain”) to 10 (“worst possible pain”). The NRS is a widely used and validated measure of pain intensity in clinical and research settings.

##### 2.3.1.1 Pain interference

Pain interference was measured using the PROMIS Pain Interference Short Form 8a (PROMIS-PI) (43). This eight-item self-report questionnaire evaluates the extent to which pain disrupts daily functioning across multiple domains, such as participation in social and work-related activities, over the past seven days. Responses are provided on a five-point Likert scale. In this study standardized T-scores were used (normal mean = 50 SD = 10, elevated mean = 60 SD = 10, high mean = 70 SD = 10), with higher scores indicating greater pain-related interference. The PROMIS-PI showed high internal reliability (Cronbach's *α* in this sample was 0.89) and has demonstrated strong validity in chronic pain populations (44, 45).

##### 2.3.1.2 Psychological variables

Anxiety symptoms experienced during the previous seven days were assessed using the PROMIS Anxiety Short Form 8a (PROMIS Anxiety) (46). This self-report instrument is comprised of eight items rated on a five-point Likert scale, ranging from “never” to “always.” Scores were converted to standardized T-scores (normal mean = 50 SD = 10, elevated mean = 60 SD = 10, high mean = 70 SD = 10), with higher values reflecting greater levels of anxiety. The PROMIS Anxiety demonstrated excellent internal consistency (Cronbach's *α* in this sample was 0.90) and has been widely validated across diverse populations, including individuals with chronic pain (45).

Pain-related cognitions and worries were assessed using the Pain Catastrophizing Scale (PCS) (47). The PCS is a 13-item self-report measure with five-point Likert-type response options ranging from “not at all” to “all the time.” The scale yields a total score, as well as subscale scores reflecting rumination, magnification, and feelings of helplessness in relation to pain. Total scores range from 0 to 52, with higher scores indicating greater levels of catastrophic thinking. In the current sample, the PCS demonstrated good internal consistency (Cronbach's *α* in this study was 0.88) and has been extensively validated in diverse populations, including individuals with chronic pain (47).

##### 2.3.1.3 Physical performance

Physical performance was assessed using the 1-min sit-to-stand test (1 min STS) (48). Participants were instructed to repeatedly stand up and sit down from a standard chair as many times as possible within a 60-second period, following a standardized protocol (48). Although this test primarily reflects lower-limb performance, it is widely used as a proxy of overall functional capacity, as it integrates muscle strength, endurance, and cardiorespiratory demands (49). Importantly, the 1 min STS has shown strong associations with broader indicators of functional status, including mobility, aerobic capacity, and activities of daily living across clinical populations, supporting its use as a pragmatic measure of global physical functioning (48). It was selected due to the absence of a single standardized measure capturing global physical function in this population.

##### 2.3.1.4 Heart rate and oxygen saturation

Physiological responses to the 1 min STS were evaluated through monitoring of heart rate (HR) and peripheral oxygen saturation (SpO₂) (48). Baseline values were recorded for 1 min immediately before the 1 min STS, a standardized resting period prior to the test. Post-test values were recorded immediately upon test completion. Changes in HR (*Δ*HR) and SpO₂ (*Δ*SpO₂) were calculated as the difference between post-test and baseline measurements, providing indices of cardiovascular and oxygenation responses to functional exertion (48, 50).

##### 2.3.1.5 Hair cortisol concentration

Hair cortisol concentration was assessed as an index of long-term hypothalamic–pituitary–adrenal axis activity (51). Hair samples were collected during the in-person study visit. A strand of hair was cut from the posterior vertex region of the scalp, as close to the scalp as possible, following established recommendations for hair cortisol sampling. For each participant, a minimum of 10 mg of hair was obtained (52). The proximal 3 cm segment of hair was selected for analysis, corresponding to an estimated three-month period of cumulative cortisol secretion, based on an average hair growth rate of approximately 1 cm per month (51, 52). Samples were immediately wrapped in aluminum foil, labeled to indicate the scalp end, and stored at room temperature until shipment. Hair samples were sent to the Department of Psychology at Technische Universität Dresden (TU Dresden) in Germany for biochemical analysis. Cortisol extraction and quantification were performed using liquid chromatography coupled with tandem mass spectrometry (LC–MS/MS), a highly sensitive and specific method for hair cortisol assessment (51, 52). All analyses were conducted according to standardized laboratory protocols at TU Dresden. Hair cortisol values were returned to the study team for subsequent statistical analyses.

##### 2.3.1.6 Resting-state cortical hemodynamic activity

Resting-state cortical hemodynamic activity was assessed using functional near-infrared spectroscopy (fNIRS). Continuous-wave fNIRS data were acquired using a multi-channel system with long-separation source–detector pairs, sensitive to cortical hemodynamic changes, as well as short-separation channels to account for superficial physiological signals (53). The Optical Imaging System (CW7, TechEn Inc., Milford, MA, USA) was used to record time courses of oxyhemoglobin (HbO) and deoxyhemoglobin (HbR) concentrations at a sampling rate of 25 Hz. Consistent with prior resting-state fNIRS studies, analyses focused on HbO due to its higher signal-to-noise ratio and greater sensitivity to regional cerebral blood flow changes. In addition, resting-state fNIRS provides a non-invasive approach to capture intrinsic cortical activity and baseline functional organization, which may be altered in chronic pain conditions even in the absence of task demands. Concentration changes were calculated values following standard preprocessing procedures, including (1) motion artifact correction, (2) temporal band-pass filtering to remove physiological noise and slow signal drift, and (3) regression of superficial signals using short-separation channels (54–57).

For the purposes of this study, fNIRS outcomes were defined at the level of six predefined cortical regions of interest (ROIs). ROIs were selected based on their established involvement in pain processing and modulation. These regions have been consistently implicated in the top-down and bottom-up mechanisms underlying pain perception and regulation, making them particularly relevant for investigating potential cortical alterations in CPSP (58–60). They included the left lateral prefrontal cortex (PFC), left medial PFC, right medial PFC, right lateral PFC, left somatosensory cortex (SMC), and right SMC (58–60). Signals from all valid channels within each ROI were averaged to obtain a single representative measure per region, thereby reducing channel-level noise and improving the stability and interpretability of region-level estimates. Resting-state HbO was selected as the primary fNIRS-derived variable, given its sensitivity to regional cerebral perfusion and baseline cortical activity as well as its robustness in detecting between-group differences in clinical populations (60). For each ROI, mean HbO values were calculated across the resting-state recording period and used as indices of baseline cortical hemodynamic activity.

Resting-state fNIRS data were acquired during a continuous 10 min recording period in a darkened room. Participants were instructed to remain seated, awake, and as still as possible, with eyes open throughout the recording. No cognitive or motor tasks were administered, allowing for the assessment of intrinsic (task-free) cortical hemodynamic activity. Participants were asked to avoid movement and deliberate mental strategies during data acquisition.

### 2.4 Procedure

Participants were recruited through academic medical centers and community-based settings, including institutional recruitment platforms, posted advertisements, and online outreach focused on the two medical centers in the northeast region of the United States. Individuals who expressed interest were initially screened for eligibility through a brief remote screening call. Eligible participants were then scheduled for an in-person study visit. Upon arrival, written informed consent was obtained prior to the initiation of any study procedures. Data collection was conducted during a single laboratory visit and followed a standardized protocol. Participants completed demographic, medical, and psychosocial questionnaires, followed by hair specimen collection, functional testing, and neuroimaging. All self-report data were collected using secure electronic data capture systems. Study staff were present throughout the visit to ensure protocol adherence and participant safety.

### 2.5 Data analysis

Descriptive statistics were used to summarize participant characteristics separately for AYAs with CPSP and healthy controls. Means and standard deviations (SD) or medians and interquartile ranges (IQR) were reported for continuous variables. Frequencies and percentages were reported for categorical variables. Distributional properties of all continuous variables were examined visually and analytically to assess normality, plausibility of values, and the presence of outliers.

Hair cortisol concentration was inspected for skewness and log-transformed when the variable showed significant positive skewness. Physiological responses to functional activity were defined as the change in values between baseline and immediately post–1 min STS for *Δ*HR and *Δ*SpO₂. Mean HbO values were computed by averaging across all valid channels within each of the six predefined ROIs.

To address the first aim, independent-samples *t* tests were used for approximately normally distributed outcomes, whereas Mann–Whitney *U*-tests were applied when normality assumptions were not met. Effect sizes were calculated for all between-group comparisons and reported as Cohen's d, along with 95% confidence intervals, to facilitate interpretation of the magnitude and clinical relevance of observed differences. Effect sizes were interpreted using conventional thresholds, with Cohen's d values of 0.20, 0.50, and 0.80 interpreted as small, medium, and large effects, respectively (61).

To address the second aim, Pearson or Spearman correlation coefficients were applied depending on distributional characteristics. Effect sizes were quantified using correlation coefficients (Pearson's r or Spearman's *ρ*, as appropriate), with corresponding 95% confidence intervals reported. Correlation coefficients of 0.10, 0.30, and 0.50 were interpreted as small, medium, and large associations respectively (62).

To address the third, exploratory aim, hierarchical agglomerative clustering was performed using Euclidean distance and Ward's linkage method, which minimizes within-cluster variance and is well-suited for identifying homogeneous subgroups based on 1 min STS performance. The optimal number of preliminary clusters was determined based on inspection of the dendrogram, internal validity indices such as silhouette width, and clinical interpretability of the resulting phenotypes. Cluster stability was evaluated through sensitivity analyses, including comparison with k-means clustering, using the selected number of clusters.

Following cluster assignment, CPSP preliminary clusters were characterized and compared on pain interference, psychological symptoms, *Δ*HR, *Δ*SpO₂, hair cortisol concentration (log-transformed), and resting-state cortical hemodynamic activity. To avoid circularity, 1min- STS was considered a defining variable and was excluded from subsequent cluster characterization analyses. After selecting the number of clusters, they were characterized by comparing the remaining continuous variables, all expressed as *z*-scores, using nonparametric Kruskal–Wallis tests. When significant effects were observed, *post-hoc* Dunn tests with Bonferroni correction were applied.

Statistical significance was set at *p* < 0.05 for primary analyses, with an emphasis on effect sizes and confidence intervals to support interpretation. Missing data were handled using a complete-case approach. Participants with missing data in key variables required for specific analyses (e.g., clustering variables) were excluded from those analyses. The extent of missing data was low, and no imputation procedures were applied. This approach was considered appropriate given the exploratory nature of the analyses and the limited amount of missing data.

All analyses were conducted using Python (version 3.10) (63) within the Google Colab cloud-based interactive environment to ensure reproducibility. Data manipulation, cleaning, and clustering preprocessing were executed using the NumPy and pandas' libraries (64, 65). Inferential statistical modeling was implemented using the statsmodels library. Graphical representations and data visualizations were generated using Matplotlib and Seaborn (66).

## 3 Results

### 3.1 Sample characteristics

**Figure 1:**
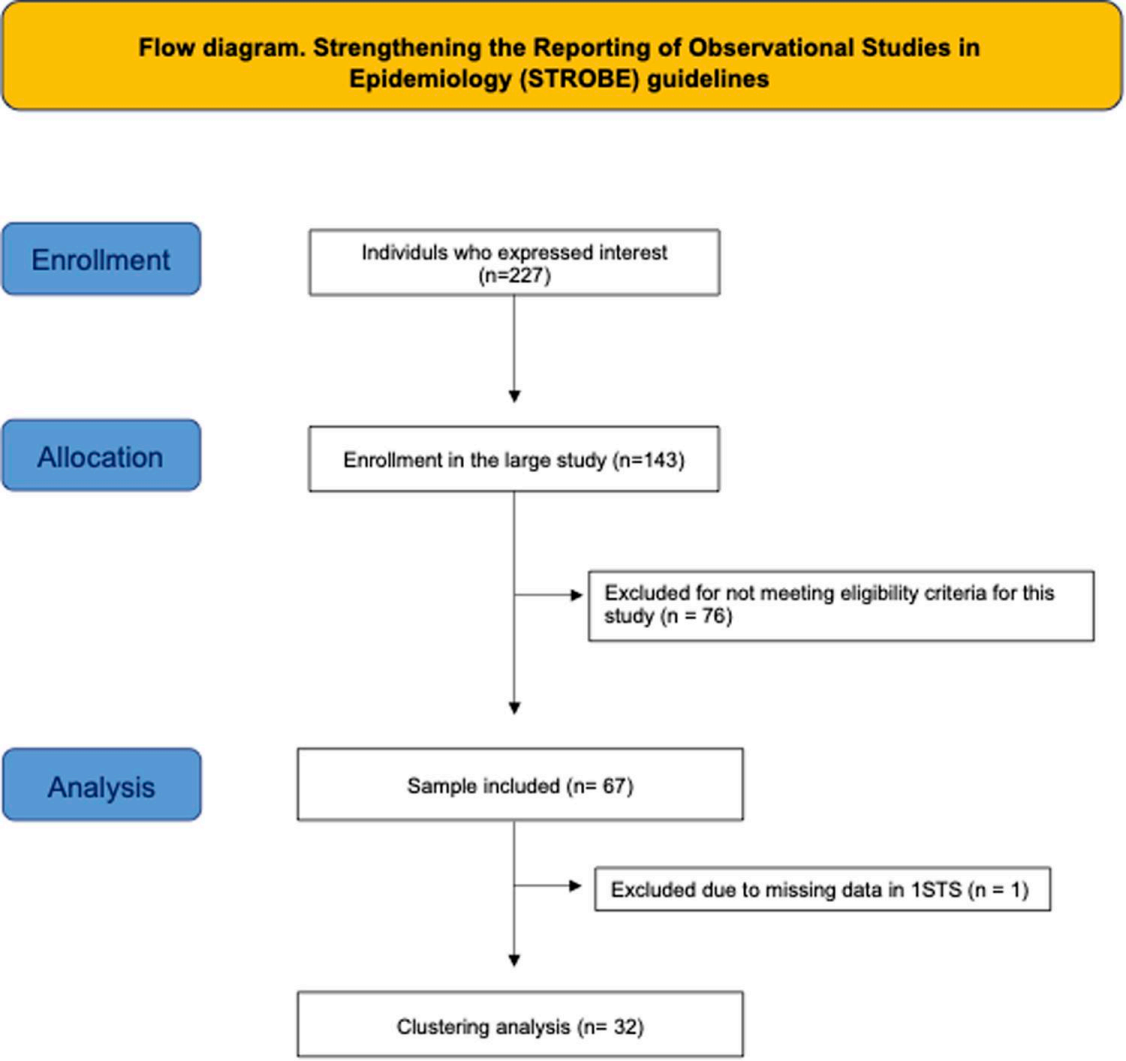
Flow diagram of participant enrollment, inclusion, and analysis according to STROBE guidelines. A total of 227 individuals were assessed for eligibility, of whom 166 were excluded from this sample for not meeting eligibility criteria. The final analytical sample included 67 participants (33 with CPSP and 34 healthy controls). One participant with CPSP was excluded from the clustering analysis due to missing 1 min sit-to-stand (1 min STS) data, resulting in a final sample of 32 individuals for the hierarchical clustering analysis.

A total of 277 individuals expressed interest in participating in the study and were assessed for eligibility. 143 were considered eligible and enrolled in the study at large. Of these 143, 67 met the eligibility criteria for this analysis and were included in the sample (see Figure 1). Data collection was conducted between 2021 and 2025 at two medical centers in the northeast region of the United States.

This sample was comprised of 67 participants, including 33 individuals with CPSP and 34 healthy controls. All variables were evaluated for normality and were found to deviate from a normal distribution. Consequently, non-parametric statistical methods were applied throughout the analyses. The sample was predominantly female (82.1%, *n* = 55) with median age of 24 years (IQR = 21.50–26.00). Participants reported moderate levels of pain catastrophizing (median = 12, IQR = 6.00–18.00) and pain interference (median = 47.90, IQR = 40.70–55.00), alongside elevated anxiety (median = 55.40, IQR = 49.40–59.40). Hair cortisol concentration showed marked dispersion (median = 1.50 IQR = 1.13–1.85 pg/mg), supporting the use of log-transformed values in subsequent analyses. 1 min STS performance was a median of 24.0 repetitions (IQR = 19.00–28.75), and physiological responses to exertion showed high variability in *Δ*HR (median = 28.00 IQR = 22.50 −35.00 bpm) and *Δ*SpO₂ (median = −1.25 IQR = −2.00–0.00) (see Table 1).

**Table 1:** Characteristics of the total sample included and by groups.

Variable	Total sample (*n* = 67) % (n)/Median (IQR)	CPSP (*n* = 33) % (n)/Median (IQR)	Heatly controls (*n* = 34) % (n)/Median (IQR)
Sex			
Female	82% (55)	93.9% (31)	70.6% (24)
Male	18% (12)	6.1% (2)	29.4% (10)
Race			
Asian or Pacific islander	10.4% (7)	9.1% (3)	11.8% (4)
Latino	6% (4)	9.1% (3)	2.9% (1)
Black or African American	7.5% (5)	3% (1)	11.8% (4)
Multiracial o Biracial	4.5% (3)	6.1% (2)	2.9% (1)
White or Caucasian	64.3% (43)	72.7% (24)	55.9% (19)
Not reported	7.5% (5)	0% (0)	14.7% (5)
Ethnicity			
Non-Hispanic	77.6% (52)	75.7% (25)	79.4% (27)
Hispanic	22.4% (15)	24.3% (8)	20.6% (7)
Age	24.00 (21.50–26.00)	25.00 (22.00–26.00)	23.00 (20.25–25.00)
Lifetime number of surgeries	–	3.5 (1–19)	–
Type of surgery			
Orthopedic	–	100% (33)	–
Subtype of surgery			
Scoliosis	–	3% (1)	–
Disc hernia	–	6% (2)	–
Rib stabilitation		3% (1)	
Hip-replacement/ Periacetabular osteotomy	–	24% (8)	–
ACL recostrution	–	27% (9)	–
Achiles repair	–	6% (2)	–
Missing data		30% (10)	
Surgery body part			
Back	–	9% (3)	–
Chest		3% (1)	
Hip	–	24% (8)	–
Knee	–	27% (9)	–
Ankle	–	6% (2)	–
Missing data	–	30% (10)	–
Pain Intensity	–	4.5 (2–7)	0.0 (0–0)
Catastrophizing (PCS)	12.00 (6.00–18.00)	15.00 (8.00–18.00)	10.50 (5.00–14.75)
Anxiety			
(PROMIS-Anxiety)	55.40 (49.40–59.40)	56.40 (49.40–60.40)	53.20 (49.40–57.40)
Pain Interference			
(PROMIS-Pain interference)	47.90 (40.70–55.00)	55.00 (51.20–58.80)	40.70 (40.70–47.90)
Hair cortisol (log)	1.50 (1.13–1.85)	1.45 (1.11–1.83)	1.55 (1.26–1.89)
Sit-to-stand test	24.00 (19.00–28.75)	21.50 (17.00–27.50)	26.00 (21.00–28.75)
*Δ*HR	28.00 (22.50 –35.00)	28.50 (22.50–35.00)	27.50 (24.00–34.50)
*Δ*SpO₂	−1.25 (−2.00–0.00)	−1.50 (−2.00–0.00)	−1.00 (−1.50–0.00)
Resting left lateral PFC	0.00 (−0.00–0.00)	0.00 (−0.00–0.06)	−0.00 (−0.00–0.00)
Resting left medial PFC	0.00 (−0.00–0.00)	0.00 (−0.00–0.03)	0.00 (−0.00–0.00)
Resting right medial PFC	0.00 (−0.00–0.00)	−0.00 (−0.00–0.07)	0.00 (0.00–0.00)
Resting right lateral PFC	0.00 (−0.00–0.00)	0.00 (−0.00–0.01)	0.00 (0.00–0.00)
Resting left SMC	0.00 (−0.00–0.00)	0.00 (−0.00–0.08)	−0.00 (−0.00–0.00)
Resting right SMC	0.00 (−0.00–0.00)	0.00 (−0.00–0.11)	−0.00 (−0.00–0.00)

IQR, Interquartile range; PCS, Pain Catastrophizing Scale, log, log-transformed values; *Δ*HR, pre-post changes during 1-STS in heart rate; *Δ*SpO₂, pre-post changes during 1-STS in peripheral oxygen saturation; PFC, prefrontal cortex; SMC, somatosensory cortex (S1).

### 3.2 Comparisons between CPSP and healthy control groups

To address the first aim, comparisons between participants with CPSP and healthy controls were analyzed. Group differences in sociodemographic characteristics were examined using Fisher's exact tests for sex and ethnicity, and a likelihood ratio chi-square test for race due to small cell counts. A significant difference in sex distribution was observed between groups, with a higher proportion of females in the CPSP group compared with healthy controls (Fisher's exact test, OR = 6.46, *p* = .023). No significant differences were observed for ethnicity (Fisher's exact test, OR = 1.23, *p* = .776). The overall racial distribution did not significantly differ between groups, although a trend toward significance was observed (likelihood ratio *χ*²(5) = 10.96, *p* = .052). On the other hand, the results revealed significant differences across select clinical, physiological, and neuroimaging variables.

#### 3.2.1 Pain interference, psychological variables, and hair cortisol

Individuals with CPSP reported statistically significantly higher levels of pain interference (*p* < 0.001, Coheńs d = 2.03) than healthy controls. However, no significant differences were observed for pain catastrophizing, anxiety symptoms, or hair cortisol concentrations (all *p* > 0.05).

#### 3.2.2 Functional performance

While no significant differences were observed for 1 min STS repetitions between groups (*p* = 0.20), participants with CPSP exhibited a statistically significantly lower *Δ*HR and *Δ*SpO₂ as compared to healthy controls (*p* < 0.001; Cohen d´s = 4.23, *p* < 0.001; Cohen d´s = 0.75, respectively). These results suggest that physiological reactivity rather than overt functional capacity differentiated CPSP participants from controls.

#### 3.2.3 Resting state fNIRS

Across resting-state regional HbO levels, no statistically significant differences were detected between groups (all p´s > 0.05).

### 3.3 Correlational analyses within the CPSP group

**Figure 2:**
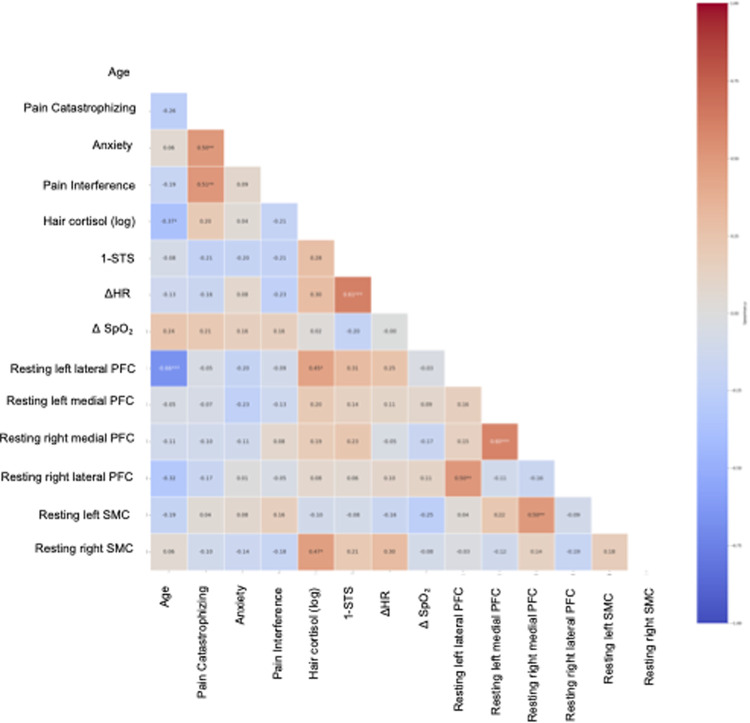
Correlation matrix of clinical, physiological, and neuroimaging variables within the CPSP group. The heatmap displays Spearman correlation coefficients among age, psychological variables (pain catastrophizing, anxiety, pain interference), physical performance (1 min sit-to-stand test; 1 min STS), physiological responses to exertion (*Δ*HR, *Δ*SpO₂), hair cortisol concentration (log-transformed), and resting-state cortical hemodynamic activity across predefined regions of interest (prefrontal and somatosensory [S1] cortices). Color intensity reflects the strength and direction of associations, with warmer colors indicating positive correlations and cooler colors indicating negative correlations. Only correlations within the CPSP group are shown.

Within the CPSP group, Spearman's correlational analyses revealed a structured pattern of associations across clinical, physical performance, hair cortisol, and resting-state neuroimaging variables (see Figure 2).

Age showed a moderate inverse correlation with hair cortisol (*ρ* = −.37, *p* < .01), indicating that younger participants tended to exhibit higher levels of hair cortisol concentration. This association may reflect age-related variability in long-term stress physiology within the CPSP group, although its interpretation should remain cautious given the sample size and cross-sectional design. Age was also strongly inversely correlated with resting-state activity in the left lateral PFC (*ρ* = −.66, *p* < .01), indicating that higher age was associated with lower resting-state values in this region. This association suggests age-related alterations in prefrontal circuits involved in cognitive control and pain modulation, which could influence regulatory strategies and top-down modulation of pain in CPSP.

At the psychological level, pain catastrophizing showed a strong positive association with anxiety symptoms (*ρ* = .50, *p* < .01) and pain interference (*ρ* = .51, *p* < .01), indicating that higher maladaptive pain-related cognitions were closely linked to greater emotional distress and functional impact.

Hair cortisol concentration showed moderate positive correlations with resting-state left lateral prefrontal cortex activity (*ρ* = .45; *p* < .01), as well as with the right SMC (*ρ* = .47; *p* < .01), indicating that higher long-term HPA axis activity was associated with greater prefrontal connectivity at rest. This pattern may reflect sustained engagement or increased regulatory demand within prefrontal circuits involved in cognitive–emotional regulation in individuals with CPSP. *Δ*HR showed a robust positive association with sit-to-stand test repetitions (*ρ* = .61, *p* < .01), reflecting coordinated autonomic and cardiopulmonary responses to functional demand.

On the other hand, several moderate positive associations were observed between resting-state measures across prefrontal and somatosensory regions. Specifically, the results showed a positive correlation between left lateral PFC and right lateral PFC (*ρ* = .50, *p* < .01), indicating greater interhemispheric coupling within lateral prefrontal regions. In addition, a strong positive association was identified between the left medial PFC and right medial PFC (*ρ* = .60, *p* < .01), reflecting coherent bilateral engagement of medial prefrontal areas at rest.

Finally, the results showed a moderate positive correlation between right medial PFC and the left SMC (*ρ* = .50, *p* < .01), suggesting increased cross-network coupling between prefrontal regulatory regions and somatosensory cortices. This pattern suggests enhanced synchronization of interhemispheric prefrontal networks involved in cognitive–affective control, alongside increased coupling between medial prefrontal and somatosensory regions, which may reflect greater top-down influence on sensory systems in individuals with CPSP.

### 3.4 Identification of preliminary functional phenotypes using hierarchical clustering

To explore whether performance-based subgroups could be identified within the CPSP group, a hierarchical agglomerative clustering approach was applied using STS as the clustering variable, expressed as a standardized z-score. Participants with valid STS data were included in this analysis (*N* = 32).

Clustering was performed using Euclidean distance and Ward's linkage methods. The optimal number of clusters was determined based on a combination of (1) visual inspection of the dendrogram, prioritizing solutions that separated major branches with clear increases in fusion distance; (2) internal validity assessed using the silhouette coefficient, evaluated for solutions ranging from *k* = 2 to *k* = 5; and (3) clinical interpretability, avoiding solutions that resulted in very small or unstable subgroups. Among the candidate solutions, a two-cluster solution (*k* = 2) demonstrated the most favorable balance of internal validity and clinical interpretability, with a high silhouette coefficient (0.621) and plausible cluster sizes. In contrast, solutions with *k* ≥ 3 tended to fragment the sample into smaller subgroups with limited clinical stability, without consistent improvement in internal validity (Table 2).

**Table 2:** Clustering characteristics.

Number of clusters	Silhouette	Groups size (n)
*K* = 2	0.621	24, 8
*K* = 3	0.550	21, 8, 3
*K* = 4	0.539	14, 8, 7, 3
*K* = 5	0.583	14, 7, 5, 3, 3

### 3.5 Exploratory description of the preliminary functional phenotype clusters

Cluster 1, termed the *Low-functioning* phenotype (*n* = 8), was characterized by poorer physical functioning, reflected by lower 1 min STS performance. Cluster 2, termed the *High-functioning* phenotype (*n* = 24), represented individuals with better functional capacity and higher 1 min STS performance. Pain intensity did not significantly differ between CPSP subgroups (*p* = 0.765). No significant differences were observed in the distribution of surgical procedures between the preliminary clusters. Similarly, no differences were found in pain location between subgroups. When pain location was categorized into upper vs. lower limb regions, the distribution was comparable across clusters. Exploratory analyses using Fisher's exact test confirmed that neither surgical type nor pain location significantly differed between CPSP subgroups (all *p* > 0.05). Preliminary cluster characteristics are presented in Table 3.

**Table 3:** Comparison of clinical, physiological, and neuroimaging variables between STS-derived clusters (z-scores).

Variable		Cluster 1 Low functioning (*n* = 8) % (n)/Mean ± SD	Cluster 2 High functioning (*n* = 24) % (n)/Mean ± SD	H	*p*-value	*p*-value adjusted
Sex						
Female		100% (8)	91.7% (22)	–	0.999*	–
Male		0% (0)	8.3% (2)	–		–
Race						
Asian or Pacific islander		0% (0)	12.5% (3)	–	0.999*^P^*	
Latino		12% (1)	8.3% (2)	–		
Black or African American		0% (0)	4.2% (1)	–		
Multiracial o Biracial		12% (1)	4.2% (1)	–		
White or Caucasian		75% (6)	70.8% (17)	–		
Not reported		0% (0)	0% (0)	–		
Ethnicity						
Non-Hispanic		62.5% (5)	79.2% (19)	–	0.378*	
Hispanic		37.5% (3)	20.8% (5)	–		
Age (z score)		0.02 ± 0.89	−0.03 ± 1.06	0.06	0.809^a^	–
Surgery subtype						
Upper extremity	Scoliosis (*n* = 1)	13% (1)	0% (0)	–	0.136	–
	Disc hernia (*n* = 2)	13% (1)	4% (1)	–	0.098	–
	Rib stabilization (*n* = 1)	0% (0)	4% (1)	–	0.357	–
Lower extremity	Hip-replacement/ Periacetabular osteotomy (*n* = 8)	37% (3)	20.8% (5)	–	0.765	–
	ACL reconstruction (*n* = 9)	37% (3)	25% (6)	–	0.368	–
	Achilles repair (*n* = 2)	0% (0)	8% (2)	–	0.097	–
Pain intensity		4.3 (1.5)	4.5 (1.2)	–	0.832	–
Catastrophizing (z score)		**−0.67** **±** **0.69**	**0.12** **±** **0.91**	**4.77**	**0**.**029**^a^	**0.028**
Anxiety (z score)		−0.48 ± 1.13	0.11 ± 0.92	2.54	0.111^a^	–
Pain Interference (z score)		−0.37 ± 0.42	0.03 ± 1.05	2.61	0.106^a^	–
Hair cortisol (log) (z score)		0.30 ± 0.79	−0.09 ± 1.08	2.40	0.122^a^	–
*Δ*HR (z score)		**0.95** **±** **0.91**	**−0.32** **±** **0.83**	**10.54**	**0**.**001**^a^	**0.001**
*Δ*SpO₂ (z score)		−0.25 ± 1.29	0.08 ± 0.90	0.50	0.478^a^	–
Resting left lateral PFC (z score)		−0.19 ± 0.57	0.06 ± 0.97	1.15	0.283^a^	–
Resting left medial PFC (z score)		−0.16 ± 0.10	0.00 ± 1.13	0.13	0.720^a^	–
Resting right medial PFC (z score)		0.03 ± 0.09	0.06 ± 1.14	0.05	0.820^a^	–
Resting right lateral PFC (z score)		−0.19 ± 0.02	0.10 ± 1.13	0.00	0.974^a^	–
Resting left SMC (z score)		−0.39 ± 0.34	−0.14 ± 1.11	0.89	0.345^a^	–
Resting right SMC (z score)		**0.59** **±** **0.91**	**−0.03** **±** **1.01**	**3.69**	**0**.**045**^a^	**0.030**

bold values indicate statistical significance;.

* *p*-values from Fisher test; *P*, *p*-values from permutation-based chi-square for multicategory;.

a *p*-values from Krustal-Wallies; H indicates the Kruskal–Wallis statistic for continuous variables only; log, log-transformed values; *Δ*HR, pre-post changes during 1-STS in heart rate; *Δ*SpO₂, pre-post changes during 1-STS in peripheral oxygen saturation, SD = Standard Desviation; PFC, prefrontal cortex; SMC, somatosensory cortex (S1).

Clusters did not differ significantly in age, sex, race or ethnicity (*p*´s > 0.05). Collectively, these results indicate that the identified preliminary functional phenotypes were not driven by sociodemographic factors. Kruskal–Wallis tests revealed significant between cluster differences in pain catastrophizing, heart rate change (*Δ*HR), and resting-state activity in the right SMC (S1) cortex (all *p*-values < 0.05; see Table 3). *post-hoc* pairwise comparisons were conducted using Dunn's test, with Bonferroni correction applied to adjust for multiple comparisons.

**Figure 3:**
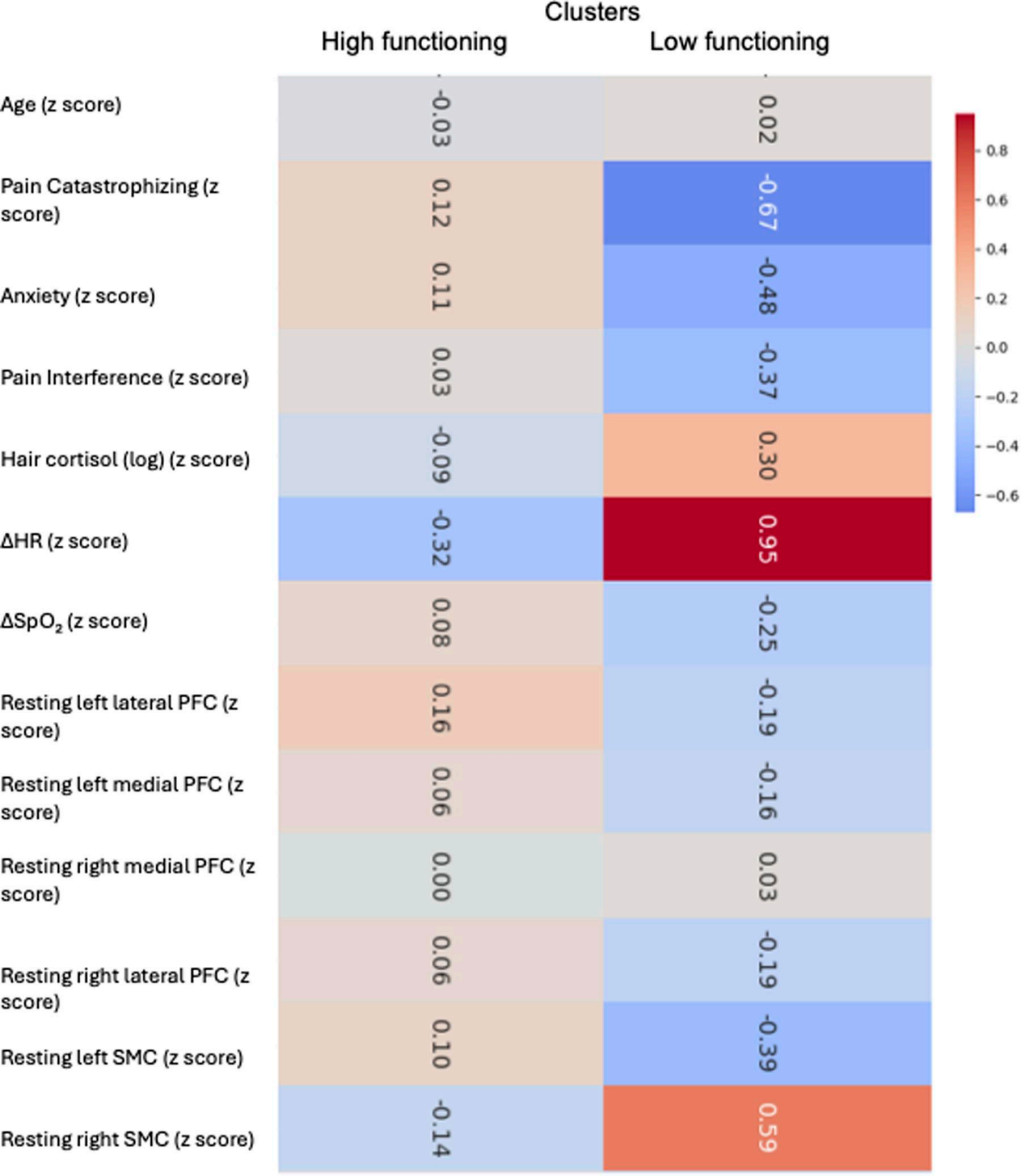
Biopsychosocial profiles of CPSP functional phenotypes identified through hierarchical clustering. Heatmap depicting standardized (z-score) values of demographic, psychological, physiological, and neuroimaging variables across the two CPSP clusters derived from 1 min sit-to-stand (1-STS) performance. The high-functioning and low-functioning phenotypes differ primarily in pain catastrophizing, autonomic reactivity to exertion (*Δ*HR), hair cortisol concentration, and resting-state somatosensory cortex activity, while showing minimal differences in age and prefrontal cortical activity. Warmer colors indicate higher standardized values and cooler colors indicate lower standardized values.

Post-hoc analyses indicated that the *Low-functioning* cluster reported higher levels of pain catastrophizing (adjusted *p* = 0.028), suggesting a greater cognitive–emotional burden related to pain. In addition, the *Low-functioning* cluster exhibited greater cardiovascular reactivity during exertion, as reflected by a significantly larger *Δ*HR following the 1 min STS task (adjusted *p* = 0.001). This pattern is consistent with reduced physiological efficiency or heightened autonomic activation in response to brief functional demands and may reflect physical deconditioning, increased interoceptive sensitivity, or elevated stress responsivity associated with persistent pain, however this should be considered exploratory (Figure 3).

Regarding resting-state cortical hemodynamic activity, no significant differences between clusters were observed in resting oxyhemoglobin levels within the left lateral prefrontal cortex, left medial prefrontal cortex, right medial prefrontal cortex, right lateral prefrontal cortex, or left SMC (S1) (all *p* > 0.28, see Table 3).

Differences were observed in the right SMC (S1), with higher resting-state activity in the *Low-functioning* cluster compared with the *High-functioning* cluster (*H* = 3.69, *p* = 0.045). This difference remained statistically significant after *post-hoc* pairwise comparisons using Dunn's test with Bonferroni correction (adjusted *p* = 0.030), indicating that functional heterogeneity captured by 1 min STS performance was specifically associated with differences in autonomic reactivity, pain-related cognitions, and select resting-state connectivity measures, rather than a global shift across all assessed domains.

## 4 Discussion

This study provides a comprehensive characterization of CPSP in AYAs by integrating psychological symptoms, objective physical performance, stress-related biomarkers, and resting-state cortical hemodynamic activity.

Consistent with our first aim, AYAs with CPSP exhibited significantly higher pain interference and altered physiological responses to functional exertion compared with healthy controls, despite comparable levels of overt physical performance. Within the CPSP group, meaningful associations emerged between psychological symptoms, stress physiology, physical functioning, and resting-state prefrontal and somatosensory activity, highlighting the interconnected nature of cognitive–emotional, physiological, and neural processes. However, these within-group associations should not be interpreted as evidence of between-group differences in cortical activity, which were not observed in this study. In an exploratory analysis, hierarchical preliminary clustering based on functional performance identified distinct CPSP phenotypes characterized by differences in pain catastrophizing, autonomic reactivity, and regional cortical activity, underscoring functional heterogeneity that is not explained by sociodemographic factors alone.

While prior work has emphasized habitual physical activity as a key protective factor in chronic pain (24, 27, 31, 36, 69–71), functional performance may represent a more proximal and clinically accessible marker of how individuals translate capacity into action under physical demand. In this context, performance-based measures such as the sit-to-stand test integrate motor capacity, autonomic regulation, and cognitive–emotional influences, offering a window into real-world functional adaptation that is not fully captured by self-reported activity levels alone (48).

Our results suggest that variability in functional performance reflects broader biopsychosocial organization, encompassing pain-related cognitions, stress-related physiological reactivity, and resting-state cortical activity. Rather than indicating fixed impairment, these patterns may index differential regulatory strategies and efficiency across individuals with CPSP, helping to explain why similar pain experiences can be associated with markedly different functional outcomes (72). From a clinical perspective, incorporating objective functional performance alongside activity-based metrics may enhance phenotyping and support more individualized, function-oriented rehabilitation approaches (73, 74).

On the other hand, when comparing AYAs with CPSP to healthy controls, a striking dissociation emerged between functional capacity and physiological reactivity. Although groups did not differ in objective physical performance as measured by the 1 min STS, individuals with CPSP showed significantly attenuated heart rate and oxygen saturation responses to exertion. This pattern suggests that persistent postsurgical pain in young individuals may be characterized less by overt reductions in functional output and more by differences in physiological responses to exertion. Importantly, no between-group differences were observed in 1 min STS performance, suggesting comparable levels of overt task execution. In this context, the observed differences in heart rate and oxygen saturation responses are more likely to reflect underlying physiological processes related to exertion rather than differences in task completion *per se* (27, 36, 75–79). At the same time, these physiological responses may also be influenced by effort-dependent and behavioral factors, including baseline physical fitness, pacing strategies, and task engagement. Therefore, these findings should be interpreted cautiously, as they may reflect a combination of physiological and behavioral contributions. Future studies incorporating direct measures of physical fitness and effort are needed to better disentangle these mechanisms.

Importantly, despite our *a priori* focus on cortical mechanisms, we did not observe significant group-level differences in resting-state fNIRS activity between CPSP participants and healthy controls. This null finding should be explicitly acknowledged and may, in part, be explained by the fact that cortical activity was assessed at rest. Resting-state measures may not adequately capture condition-specific alterations that emerge during functionally relevant states, particularly in the context of pain and movement. In this sense, the absence of group differences at rest does not provide evidence of group-level cortical differences under resting conditions but rather suggests that such differences may be more evident under task-based conditions. Notably, given the functional differences observed in this study, it is plausible that cortical alterations in CPSP are more closely linked to movement-related or performance-related processes. Therefore, future studies should consider evaluating cortical activity during motor or functionally engaging tasks, which may be more sensitive to detecting meaningful neurophysiological differences. Additionally, the heterogeneity of CPSP and the modest sample size may have further limited the detection of group-level effects, reinforcing the need for larger studies and more individualized or task-based approaches to better characterize underlying mechanisms.

Beyond group-level differences, correlational analyses within the CPSP group highlighted the central role of chronic stress physiology in shaping both functional and neural patterns (20, 21, 80, 81). Higher hair cortisol concentrations were associated with younger age, suggesting age-related differences in long-term hypothalamic–pituitary–adrenal axis activity among AYAs with CPSP (18, 21, 82). This finding may reflect developmental variability in stress regulation or differences in cumulative exposure to pain-related stressors during earlier stages of adulthood (18, 20, 22, 80).

Importantly, higher hair cortisol levels were also moderately associated with greater resting-state activity in prefrontal and somatosensory regions, particularly the lateral prefrontal cortex and right SMC (S1). These within-group associations suggest that, among individuals with CPSP, higher levels of physiological chronic stress may be related to greater baseline engagement of cortical circuits involved in cognitive–emotional regulation and somatosensory processing (18, 52, 83, 84). Rather than reflecting adaptive compensation, elevated resting-state cortical activity in this context may reflect increased regulatory demand or inefficiency at the individual level, potentially contributing to the persistence of pain and functional vulnerability in patients with CPSP (80).

To our better knowledge, this present study is among the few investigations in AYAs to examine CPSP through a phenotypic approach grounded in objective physical performance. By applying exploratory clustering methods based on 1 min STS performance, we identified distinct preliminary subgroups and phenotypes within CPSP. Preliminary functional phenotypes present differences in psychological vulnerability, autonomic reactivity, and resting-state cortical activity, capturing clinically relevant within-group variations. Importantly, the low-functioning phenotype was characterized by higher pain catastrophizing and greater cardiovascular reactivity to exertion, suggesting that reduced functional performance in CPSP may reflect a broader profile of heightened cognitive–emotional burden and physiological stress responsivity rather than isolated deficits in physical capacity (20, 80, 85, 86). The observation of increased resting-state activity in the right SMC (S1) in this phenotype is consistent with literature describing altered sensorimotor processing in persistent pain, and may reflect increased regulatory demand or inefficiency at baseline (80). From a clinical standpoint, elevated resting-state activity in somatosensory regions may signal altered sensorimotor readiness that warrants careful consideration when designing function-oriented rehabilitation strategies in young individuals with CPSP (51, 81).

Together, these findings extend existing models of CPSP by showing that not only physical activity (35), but also functional performance captures clinically relevant variations in psychological, physiological, and neurobiological profiles among young individuals with persistent postsurgical pain (7, 67, 68).

### 4.1 Limitations and future directions

Several limitations should be considered when interpreting the findings of this study. First, the observational cross-sectional design precludes causal inferences regarding the association among psychological, physiological, and neurobiological variables. Although meaningful associations were identified across domains, the temporal direction of these associations cannot be determined. Future research should prioritize longitudinal designs to examine the temporal evolution of psychological, physiological, and neurobiological changes associated with CPSP, and to identify predictors of pain persistence and recovery following surgery. Such approaches would facilitate stronger mechanistic inferences and improve clinical relevance.

Second, the sample size was modest, particularly for the clustering analyses conducted exclusively within the CPSP group, which may limit the stability of the identified clusters and the generalizability of the proposed preliminary functional phenotypes. Although statistical and clinical criteria were applied to select the optimal clustering solution, these findings should be regarded as exploratory and require replication in independent samples. Replication of the clustering analyses in larger and independent samples is also needed to evaluate the stability and reproducibility of the functional phenotypes identified in this study. External validation is a critical step before considering clinical implementation of phenotype-based stratification.

Third, the sample was predominantly composed of young adult females, which may restrict the generalizability of the results to more diverse populations in terms of sex, age, or clinical context. Additionally, participants were recruited from medical centers located in the Northeast of the United States, potentially limiting the applicability of the findings to other geographical or sociocultural settings.

Another important limitation is that cortical hemodynamic activity was assessed using resting-state fNIRS and predefined cortical regions of interest. While this approach enhances interpretability and reduces analytical complexity, it does not capture dynamic brain responses during task engagement nor activity in deeper brain structures implicated in pain processing, which require complementary neuroimaging techniques. In addition, future studies should incorporate objective measures of physical activity, movement patterns, and sedentary behavior, and should assess cortical and autonomic responses during functional or effort-based tasks. These approaches may provide more ecologically valid insights into the interaction between physical performance, physiological reactivity, and brain function.

One important limitation of this study is the relatively small sample size, particularly within the CPSP group, in relation to the complexity of the analyses performed. Specifically, the use of hierarchical clustering in a limited sample may affect the stability, reproducibility, and generalizability of the identified phenotypes. Therefore, the clustering results should be interpreted with caution and considered exploratory in nature. While the identified patterns provide potentially meaningful insights into heterogeneity within CPSP, they may be sensitive to sample-specific characteristics. Future studies with larger and independent samples are needed to evaluate the robustness of these clusters, formally assess their stability, and determine their replicability across populations.

Several potential confounding factors should be considered when interpreting the present findings. First, baseline physical fitness was not directly assessed using objective measures, which may have influenced both physical activity levels and neurophysiological outcomes. As a result, it is difficult to disentangle whether the observed differences are attributable to habitual physical activity *per se* or to underlying fitness levels. Also, variability in the type and timing of surgery was not fully controlled for in the analyses. Given that surgical characteristics (e.g., invasiveness, recovery trajectory, and time since surgery) may influence pain persistence and functional recovery, this heterogeneity could have contributed to variability within the CPSP group.

An additional limitation relates to the use of the 1 min sit-to-stand test as a proxy for overall physical functioning. Although this measure provides a practical and objective assessment of functional performance, it primarily reflects lower-limb strength and endurance, and may not fully capture global physical functioning. As such, its use may introduce bias, particularly in individuals whose pain location or functional limitations are not predominantly lower-limb related. Future studies should incorporate a broader range of functional assessments to more comprehensively capture multidimensional aspects of physical functioning.

Furthermore, variability in time since surgery was not fully accounted for in the analyses and may represent an important source of heterogeneity. Differences in recovery stage may influence physiological responses to exertion, functional performance, and pain-related outcomes. As a result, the observed findings may partially reflect differences in postoperative recovery trajectories rather than stable characteristics of CPSP. Future research should more explicitly control for time since surgery to better isolate these effects.

Although pain intensity was assessed, it was not included as a covariate in the analyses. Future studies should consider accounting for variability in pain severity, as it may influence cortical and physiological responses.

Finally, although multiple clinical domains were integrated, other potentially relevant factors—such as objective measures of daily physical activity, sleep quality, or contextual and environmental variables—were not assessed and may further contribute to functional heterogeneity in CPSP.

## 5 Conclusions

These findings suggest that CPSP in AYAs is associated with distinct patterns of perceived functional impact and physiological reactivity, rather than with uniform deficits in physical capacity or baseline cortical activity. Together, these results support an integrated biopsychosocial framework in which psychological factors, stress-related physiology, and cortical regulation interact to shape functional outcomes following surgery. Within this framework, a low-functioning phenotype was identified, marked by higher pain catastrophizing, greater cardiovascular reactivity to exertion, and increased resting-state activity in the right SMC (S1). These patterns highlight the heterogeneity of CPSP and suggest that objective physical performance may provide a clinically meaningful lens through which to identify subgroups with differing biopsychosocial profiles, potentially informing more individualized and function-oriented approaches to assessment and rehabilitation.

## Data Availability

The raw data supporting the conclusions of this article will be made available by the authors, without undue reservation.

## References

[1] Murray CB de la Vega R Murphy LK Kashikar-Zuck S Palermo TM. The prevalence of chronic pain in young adults: a systematic review and meta-analysis. Pain. (2022) 163(9):e972–e84. 10.1097/j.pain.0000000000002541 34817439

[2] Hestbaek L Leboeuf-Yde C Kyvik KO Manniche C. The course of low back pain from adolescence to adulthood: eight-year follow-up of 9600 twins. Spine (Phila Pa 1976). (2006) 31(4):468–72. 10.1097/01.brs.0000199958.04073.d9 16481960

[3] Tham SW Murray CB Law EF Slack KE Palermo TM. The impact of the COVID-19 pandemic on pain and psychological functioning in young adults with chronic pain. Pain. (2022) 163(10):e1095–e101. 10.1097/j.pain.0000000000002618 35413028 PMC9470785

[4] Murray CB Li R Kashikar-Zuck S Zhou C Palermo TM. Adolescent predictors of young adult pain and health outcomes: results from a 6-year prospective follow-up study. Pain. (2025) 166(1):42–51. 10.1097/j.pain.0000000000003308 38916525 PMC12169839

[5] Phillips N Brown BT Jones MP Magson N Beynon A Swain MS. The association between bullying victimization and back pain in young people: a systematic literature review and meta-analysis. Pain. (2025) 166(3):502–10. 10.1097/j.pain.0000000000003398 39297723

[6] Rosenbloom BN Frederiksen SD Wang V Birnie KA Park CS Gordon G Prevalence of and recommendation for measuring chronic postsurgical pain in children: an updated systematic review and meta-analysis. Reg Anesth Pain Med. (2025) 50(2):132–43. 10.1136/rapm-2024-105697 39909546 PMC11804871

[7] Rabbitts JA Fisher E Rosenbloom BN Palermo TM. Prevalence and predictors of chronic postsurgical pain in children: a systematic review and meta-analysis. J Pain. (2017) 18(6):605–14. 10.1016/j.jpain.2017.03.007 28363861 PMC5457338

[8] Ellyson A Powelson E Groenewald C Rabbitts J. Healthcare utilization and costs among pediatric patients with chronic postsurgical pain after major musculoskeletal surgery. Paediatr Anaesth. (2022) 32(4):577–8. 10.1111/pan.14402 35075715 PMC9269163

[9] Rabbitts J Groenewald C. Epidemiology of pediatric surgery in the United States. Paediatr Anaesth. (2020) 30(10):1083–90. 10.1111/pan.13993 32777147 PMC7891905

[10] Rabbitts J Groenewald C Moriarty J Flick R. Epidemiology of ambulatory anesthesia for children in the United States: 2006 and 1996. Anesth Analg. (2010) 111(4):1011–5. 10.1213/ANE.0b013e3181ee8479 20802051

[11] Ceniza-Bordallo G Gómez Fraile A Martín-Casas P Rabbitts J Li R Palermo T Prevalence, pain trajectories, and presurgical predictors for chronic postsurgical pain in a pediatric sample in Spain with a 24-month follow-up. Pain. (2025) 166(1):112–22. 10.1097/j.pain.0000000000003330 39047258 PMC11856900

[12] Ceniza-Bordallo G Fraile AG López-de-Uralde-Villanueva I Rabbitts JA Li R Palermo TM Chronic postsurgical pain in young children: prevalence, pain trajectories and physical and psychological prognostic factors. J Pain. (2025) 34:105476. 10.1016/j.jpain.2025.105476 40562263 PMC12282272

[13] Twiddy H Hanna J Haynes L. Growing pains: understanding the needs of emerging adults with chronic pain. Br J Pain. (2017) 11(3):108–18. 10.1177/2049463717709641 28785407 PMC5521350

[14] Lynch Milder M Ward S Bazier A Stumpff J Tsai Owens M Williams A. The health care transition needs of adolescents and emerging adults with chronic pain: a narrative review. J Clin Psychol Med Settings. (2023) 31(1):26–36. 10.1007/s10880-023-09966-0 37358678

[15] Feinstein AB Sturgeon JA Darnall BD Dunn AL Rico T Kao MC The effect of pain catastrophizing on outcomes: a developmental perspective across children, adolescents, and young adults with chronic pain. J Pain. (2017) 18(2):144–54. 10.1016/j.jpain.2016.10.009 27825857 PMC5291801

[16] Feinstein AB Brown K Dunn AL Neville AJ Sokol O Poupore-King H Where do we start? Health care transition in adolescents and young adults with chronic primary pain. Pain. (2025) 166(2):236–42. 10.1097/j.pain.0000000000003324 38981053

[17] Murray CB Murphy LK Jordan A Owens MT McLeod D Palermo TM. Healthcare transition among young adults with childhood-onset chronic pain: a mixed methods study and proposed framework. J Pain. (2022) 23(8):1358–70. 10.1016/j.jpain.2022.02.010 35301116 PMC12169605

[18] Nelson S Burns M McEwen B Borsook D. Stressful experiences in youth: “set-up” for diminished resilience to chronic pain. Brain Behav Immun Health. (2020) 5:100095. 10.1016/j.bbih.2020.100095 34589863 PMC8474662

[19] McCormick CM Mathews IZ. Adolescent development, hypothalamic-pituitary-adrenal function, and programming of adult learning and memory. Prog Neuropsychopharmacol Biol Psychiatry. (2010) 34(5):756–65. 10.1016/j.pnpbp.2009.09.019 19782715

[20] James KA Stromin JI Steenkamp N Combrinck MI. Understanding the relationships between physiological and psychosocial stress, cortisol and cognition. Front Endocrinol. (2023) 14:1085950. 10.3389/fendo.2023.1085950 PMC10025564 36950689

[21] Martikainen S Pesonen AK Lahti J Heinonen K Pyhälä R Tammelin T Physical activity and hypothalamic-pituitary-adrenocortical axis function in adolescents. Psychoneuroendocrinology. (2014) 49:96–105. 10.1016/j.psyneuen.2014.06.023 25068769

[22] Naya CH Zink J Huh J Dunton GF Belcher BR. Examining the same-day relationship between morning cortisol after awakening, perceived stress in the morning, and physical activity in youth. Stress. (2021) 24(3):338–47. 10.1080/10253890.2020.1804852 32840163 PMC7904965

[23] Costici E De Salvatore S Oggiano L Sessa S Curri C Ruzzini L The impact of physical activity on adolescent low back pain: a systematic review. J Clin Med. (2024) 13(19):5760. 10.3390/jcm13195760 39407820 PMC11477100

[24] Hill L Roofigari N Faraz M Popov J Moshkovich M Figueiredo M Physical activity in pediatric inflammatory bowel disease: a scoping review. Pediatr Exerc Sci. (2024) 36(1):44–56. 10.1123/pes.2022-0078 37487582

[25] Lodovica LMF Francesca M Paolo P Gabriele T Anselmo C Caleb D The effects of different levels of sports activity on health-related quality of life and lifestyle habits in high school Italian students. Eur J Pediatr. (2024) 183(9):4041-4048. 10.1007/s00431-024-05661-w 38955848 PMC11322414

[26] McGranahan MJ O'Connor PJ. Influence of regular physical activity on sleep. Curr Top Behav Neurosci. (2024) 67:309–28. 10.1007/7854_2024_503 39080238

[27] Ozdemir BC Savci S Tanriverdi A Ozcan Kahraman B Isguder R Makay B Determinants of physical activity level in children and adolescents with juvenile idiopathic arthritis. Z Rheumatol. (2024) 84(Suppl 1):71–7. 10.1007/s00393-023-01340-7 37010629

[28] Galmés-Panadés AM Vidal-Conti J. Association between physical fitness and low back pain: the pepe cross-sectional study. Children. (2022) 9(9):350. 10.3390/children9091350 36138660 PMC9498200

[29] Kolb S Burchartz A Krause L Klos L Schmidt S Woll A Physical activity and recurrent pain in children and adolescents in Germany-results from the MoMo study. Children. (2022) 9(11):1645. 10.3390/children9111645 36360373 PMC9689024

[30] de Aguiar Greca JP Korff T Ryan J. Associations between children’s physical activity, pain and injuries. Percept Mot Skills. (2021) 128(5):1959–74. 10.1177/00315125211028455 34187240 PMC8414821

[31] Tomschi F Kieckbusch L Zachow J Hilberg T. Does exercise-induced hypoalgesia depend on exercise duration? Biology. (2023) 12(2):222. 10.3390/biology12020222 36829500 PMC9953562

[32] Lemes Í R Oliveira CB Silva GCR Pinto RZ Tebar WR Christofaro DG. Association of sedentary behavior and early engagement in physical activity with low back pain in adolescents: a cross-sectional epidemiological study. Eur Spine J. (2022) 31(1):152–8. 10.1007/s00586-021-07004-x 34586504

[33] Tomschi F Lieverkus D Hilberg T. Exercise-induced hypoalgesia (EIH) in response to different exercise intensities. Eur J Appl Physiol. (2022) 122(10):2213–22. 10.1007/s00421-022-04997-1 35809091 PMC9463310

[34] Kędra A Plandowska M Kędra P Czaprowski D. Physical activity and low back pain in children and adolescents: a systematic review. Eur Spine J. (2021) 30(4):946–56. 10.1007/s00586-020-06575-5 32845380

[35] Rabbitts J Holley A Zhou C Chen L. Physical activity as a predictor of chronic pain following pediatric spinal surgery. Clin J Pain. (2021) 37(3):186–93. 10.1097/AJP.0000000000000903 33273273 PMC7867602

[36] Lukkahatai N Ong IL Benjasirisan C Saligan LN. Brain-Derived neurotrophic factor (BDNF) as a marker of physical exercise or activity effectiveness in fatigue, pain, depression, and sleep disturbances: a scoping review. Biomedicines. (2025) 13(2):332. 10.3390/biomedicines13020332 40002745 PMC11853410

[37] Lepping RJ Hoffart CM Bruce AS Taylor JM Mardis NJ Lim SL Pediatric neural changes to physical and emotional pain after intensive interdisciplinary pain treatment: a pilot study. Clin J Pain. (2023) 40(11):665–72. 10.1097/AJP.0000000000001237 39514716

[38] Senba E Kami K. Exercise therapy for chronic pain: how does exercise change the limbic brain function? Neurobiol Pain. (2023) 14:100143. 10.1016/j.ynpai.2023.100143 38099274 PMC10719519

[39] Tong H Maloney TC Payne MF King CD Ting TV Kashikar-Zuck S Processing of pain by the developing brain: evidence of differences between adolescent and adult females. Pain. (2022) 163(9):1777–89. 10.1097/j.pain.0000000000002571 35297790 PMC9391252

[40] von Elm E Altman DG Egger M Pocock SJ Gøtzsche PC Vandenbroucke JP. The strengthening the reporting of observational studies in epidemiology (STROBE) statement: guidelines for reporting observational studies. PLoS Med. (2007) 4(10):e296. 10.1371/journal.pmed.0040296 17941714 PMC2020495

[41] Lunde CE Fisher E Donovan E Serbic D Sieberg CB. Cutting the cord? Parenting emerging adults with chronic pain. Paediatr Neonatal Pain. (2022) 4(3):136–47. 10.1002/pne2.12072 36188158 PMC9485821

[42] Schug SA Lavand'homme P Barke A Korwisi B Rief W Treede R-D The IASP classification of chronic pain for ICD-11: chronic postsurgical or posttraumatic pain. Pain. (2019) 160(1):45–52. 10.1097/j.pain.0000000000001413 30586070

[43] Varni J Stucky B Thissen D DeWitt E Irwin D Lai J PROMIS Pediatric pain interference scale: an item response theory analysis of the pediatric pain item bank. J Pain. (2010) 11(11):1109–19. 10.1016/j.jpain.2010.02.005 20627819 PMC3129595

[44] Ceniza-Bordallo G Fraile A Martín-Casas P López-de-Uralde-Villanueva I. Validity and reliability of Spanish PROMIS pediatric pain interference short form. J Pediatr Nurs. (2022) 6(66):79–85. 10.1016/j.pedn.2022.05.015 35687928

[45] Jacobson CJ Farrell JE Kashikar-Zuck S Seid M Verkamp E Dewitt EM. Disclosure and self-report of emotional, social, and physical health in children and adolescents with chronic pain–a qualitative study of PROMIS pediatric measures. J Pediatr Psychol. (2013) 38(1):82–93. 10.1093/jpepsy/jss099 23027719 PMC3547235

[46] Irwin DE Stucky B Langer MM Thissen D Dewitt EM Lai JS An item response analysis of the pediatric PROMIS anxiety and depressive symptoms scales. Qual Life Res. (2010) 19(4):595–607. 10.1007/s11136-010-9619-3 20213516 PMC3158603

[47] Sullivan M Bishop S Pivik J. The pain catastrophizing scale: development and validation. Psychol Assess. (1995) 7:524–32. 10.1037/1040-3590.7.4.524

[48] Bohannon RW Crouch R. 1-Minute Sit-to-Stand test: systematic review of procedures, performance, and clinimetric properties. J Cardiopulm Rehabil Prev. (2019) 39(1):2–8. 10.1097/HCR.0000000000000336 30489442

[49] Vilarinho R Montes AM Noites A Silva F Melo C. Reference values for the 1-minute sit-to-stand and 5 times sit-to-stand tests to assess functional capacity: a cross-sectional study. Physiotherapy. (2024) 124:85–92. 10.1016/j.physio.2024.01.004 38875841

[50] Bohannon RW Crouch RH. Two-Minute step test of exercise capacity: systematic review of procedures, performance, and clinimetric properties. J Geriatr Phys Ther. (2019) 42(2):105–12. 10.1519/JPT.0000000000000164 29210933

[51] Stalder T Kirschbaum C. Analysis of cortisol in hair–state of the art and future directions. Brain Behav Immun. (2012) 26(7):1019–29. 10.1016/j.bbi.2012.02.002 22366690

[52] Sauvé B Koren G Walsh G Tokmakejian S Van Uum SH. Measurement of cortisol in human hair as a biomarker of systemic exposure. Clin Invest Med. (2007) 30(5):E183–91. 10.25011/cim.v30i5.2894 17892760

[53] Wyser D Mattille M Wolf M Lambercy O Scholkmann F Gassert R. Short-channel regression in functional near-infrared spectroscopy is more effective when considering heterogeneous scalp hemodynamics. Neurophotonics. (2020) 7(3):035011. 10.1117/1.NPh.7.3.035011 33029548 PMC7523733

[54] Jahani S Setarehdan SK Boas DA Yücel MA. Motion artifact detection and correction in functional near-infrared spectroscopy: a new hybrid method based on spline interpolation method and savitzky-golay filtering. Neurophotonics. (2018) 5(1):015003. 10.1117/1.NPh.5.1.015003 29430471 PMC5803523

[55] Gao L Wei Y Wang Y Wang G Zhang Q Zhang J Hybrid motion artifact detection and correction approach for functional near-infrared spectroscopy measurements. J Biomed Opt. (2022) 27(2):025003. 10.1117/1.JBO.27.2.025003 35212200 PMC8871689

[56] Bergmann T Vakitbilir N Gomez A Islam A Stein KY Sainbhi AS Artifact management for cerebral near-infrared spectroscopy signals: a systematic scoping review. Bioengineering. (2024) 11(9):933. 10.3390/bioengineering11090933 39329675 PMC11428271

[57] Sato T Nambu I Takeda K Aihara T Yamashita O Isogaya Y Reduction of global interference of scalp-hemodynamics in functional near-infrared spectroscopy using short distance probes. Neuroimage. (2016) 141:120–32. 10.1016/j.neuroimage.2016.06.054 27374729

[58] Kropf E Syan SK Minuzzi L Frey BN. From anatomy to function: the role of the somatosensory cortex in emotional regulation. Braz J Psychiatry. (2019) 41(3):261–9. 10.1590/1516-4446-2018-0183 30540029 PMC6794131

[59] Patel M Hasoon J Diez Tafur R Lo Bianco G Abd-Elsayed A. The impact of chronic pain on cognitive function. Brain Sci. (2025) 15(6):559. 10.3390/brainsci15060559 40563731 PMC12190199

[60] Fiúza-Fernandes J Pereira-Mendes J Esteves M Radua J Picó-Pérez M Leite-Almeida H. Common neural correlates of chronic pain - A systematic review and meta-analysis of resting-state fMRI studies. Prog Neuropsychopharmacol Biol Psychiatry. (2025) 138:111326. 10.1016/j.pnpbp.2025.111326 40086716

[61] Schäfer T Schwarz MA. The meaningfulness of effect sizes in psychological research: differences between sub-disciplines and the impact of potential biases. Front Psychol. (2019) 10:813. 10.3389/fpsyg.2019.00813 31031679 PMC6470248

[62] Schober P Boer C Schwarte LA. Correlation coefficients: appropriate use and interpretation. Anesth Analg. (2018) 126(5):1763–8. 10.1213/ANE.0000000000002864 29481436

[63] Van Rossum G Drake F. The python language reference manual (Python Manual) (2009).

[64] Harris CR Millman KJ van der Walt SJ Gommers R Virtanen P Cournapeau D Array programming with NumPy. Nature. (2020) 585(7825):357–62. 10.1038/s41586-020-2649-2 32939066 PMC7759461

[65] team Tpd. pandas-dev/pandas: Pandas (v2.0.0). Zenodo. Available online at: 10.5281/zenodo.3509134

[66] Waskom ML. Seaborn: statistical data visualization. J Open Source Softw. (2023) 26(4):3021. 10.21105/joss.03021

[67] Rabbitts J Palermo T Lang E. A conceptual model of biopsychosocial mechanisms of transition from acute to chronic postsurgical pain in children and adolescents. J Pain Res. (2020) 13:3071–80. 10.2147/JPR.S239320 33262642 PMC7699440

[68] Rabbitts J Kain Z. Perioperative care for adolescents undergoing major surgery: a biopsychosocial conceptual framework. Anesth Analg. (2019) 129(4):1181–4. 10.1213/ANE.0000000000004048 30720491 PMC6867702

[69] Ceniza-Bordallo G Sanchez-Rodriguez E Roman-Juan J Jensen M Miro J. The role of physical activity in children and adolescents with chronic pain: the moderating effects of psychological symptoms and sleep difficulties. Pain. (2026); In press. 10.1097/j.pain.0000000000003997 42093187

[70] Nascimento Leite M Kamper SJ O'Connell NE Michaleff ZA Fisher E Viana Silva P Physical activity and education about physical activity for chronic musculoskeletal pain in children and adolescents. Cochrane Database Syst Rev. (2023) 7(7):Cd013527. 10.1002/14651858.CD013527.pub2 37439598 PMC10339856

[71] Larrinaga-Undabarrena A Río X Sáez I Angulo-Garay G Aguirre-Betolaza AM Albisua N Physical activity levels and sleep in schoolchildren (6–17) with and without school sport. Int J Environ Res Public Health. (2023) 20(2):1263. 10.3390/ijerph20021263 36674025 PMC9859001

[72] Li R Groenewald C Tham SW Rabbitts JA Ward TM Palermo TM. Influence of chronotype on pain incidence during early adolescence. Pain. (2024) 165(11):2595–605. 10.1097/j.pain.0000000000003271 38809249 PMC11817718

[73] Ceniza-Bordallo G Simons L Martin-Casas P Sieberg C. Long-term predictive validity of the pediatric pain screening tool for chronic postsurgical pain and pain-related quality of life impairment: associations with risk stratification groups in Spain. J Pain. (2026) 41:106221. 10.1016/j.jpain.2026.106221 41687701

[74] Ceniza-Bordallo G Guerra-Armas J Flores-Cortes M Bermudez-Ramirez S. Multimodal physiotherapist intervention for physical and psychological functioning in children with chronic pain: insights from risk stratification with the pediatric pain screening tool and recommendation to clinical practice. J Clin Med. (2025) 14(11):3629. 10.3390/jcm14113629 40507390 PMC12156140

[75] Helvacı G Tayhan F. Determinants and relationships of digital addiction, diet quality, and physical activity in adolescents. Front Public Health. (2025) 13:1654322. 10.3389/fpubh.2025.1654322 40969654 PMC12440890

[76] de Koning L Warnink-Kavelaars J van Rossum M Limmen S Van der Looven R Muiño-Mosquera L Physical activity and physical fitness in children with heritable connective tissue disorders. Front Pediatr. (2023) 11:1057070. 10.3389/fped.2023.1057070 37009265 PMC10065825

[77] Pigott T McPeak A de Chastelain A DeMayo M Rasic N Rayner L Changes in brain GABA and glutamate and improvements in physical functioning following intensive pain rehabilitation in youth with chronic pain. J Pain. (2023) 24(7):1288–97. 10.1016/j.jpain.2023.02.027 36966034

[78] González-Gálvez N Vaquero-Cristobal R Maciá-Andreu MJ García-Tascon M Soler-Marín A Gallardo-Guerrero AM. Influence of physical fitness components on personality factors and risk perception of children and adolescents: a cross-sectional study. BMJ Open. (2023) 13(12):e071995. 10.1136/bmjopen-2023-071995 PMC10728990 38072471

[79] Tucker S Heneghan NR Gardner A Rushton A Alamrani S Soundy A. Factors influencing participation in physical activity, sports, and exercise in children and adolescents with spinal pain or spinal conditions: a systematic review and meta-ethnography. Behav Sci. (2023) 13(6):486. 10.3390/bs13060486 37366738 PMC10295783

[80] Moreland M Curry C Wang A Vansickel M Wu Z Sieberg CB. Somatosensory and prefrontal cortex activity relates to emotional outcomes and hair cortisol concentration in chronic postsurgical pain. Sci Rep. (2025) 15(1):16304. 10.1038/s41598-025-00685-0 40348822 PMC12065869

[81] Bates R Salsberry P Ford J. Measuring stress in young children using hair cortisol: the state of the science. Biol Res Nurs. (2017) 19(5):499–510. 10.1177/1099800417711583 28617035 PMC6775674

[82] Engel ML Gunnar MR. The development of stress reactivity and regulation during human development. Int Rev Neurobiol. (2020) 150:41–76. 10.1016/bs.irn.2019.11.003 32204834

[83] Russell E Koren G Rieder M Van Uum S. Hair cortisol as a biological marker of chronic stress: current status, future directions and unanswered questions. Psychoneuroendocrinology. (2012) 37(5):589–601. 10.1016/j.psyneuen.2011.09.009 21974976

[84] Meyer JS Novak MA. Minireview: hair cortisol: a novel biomarker of hypothalamic-pituitary-adrenocortical activity. Endocrinology. (2012) 153(9):4120–7. 10.1210/en.2012-1226 22778226 PMC3423616

[85] Melchior M Kuhn P Poisbeau P. The burden of early life stress on the nociceptive system development and pain responses. Eur J Neurosci. (2022) 55(9-10):2216–41. 10.1111/ejn.15153 33615576

[86] Lindell M Grimby-Ekman A. Stress, non-restorative sleep, and physical inactivity as risk factors for chronic pain in young adults: a cohort study. PLoS One. (2022) 17(1):e0262601. 10.1371/journal.pone.0262601 35061825 PMC8782303

